# Designing a CXCL8-hsa chimera as potential immunmodulator of the tumor micro-environment

**DOI:** 10.3389/fimmu.2025.1539733

**Published:** 2025-03-07

**Authors:** Tanja Gerlza, Christina Trojacher, Thomas Fuchs, Aid Atlic, Roland Weis, Tiziana Adage, Andreas J. Kungl

**Affiliations:** ^1^ Karl-Franzens-University Graz, Institute of Pharmaceutical Sciences, Pharmaceutical Chemistry, Graz, Austria; ^2^ Medical University Graz, Otto Loewi Research Center, Graz, Austria; ^3^ Validogen GmbH, Graz, Austria; ^4^ Antagonis Biotherapeutics GmbH, Graz, Austria

**Keywords:** chemokines, fluorescence spectroscopy, glycosaminoglycans, heparan sulfate, surface plasmon resonance, Boyden chamber

## Abstract

**Introduction:**

CXCL8, belonging to inflammatory chemokines, is expressed by various cell types and plays a key role in leukocyte trafficking during infections, inflammatory processes, tissue injury and tumor progression. Chemokines interact not only with G-protein coupled receptors but also with glycosaminoglycans (GAGs), which are polyanionic linear polysaccharides. Chemokine-GAG interactions are critical for creating localized concentration gradients, protecting chemokines from degradation, and maintaining their efficacy *in vivo*.

**Methods:**

We have previously engineered a CXCL8-based dominant-negative decoy (“PA401”) with strongly increased GAG binding affinity combined with complete GPCR knockout, which was originally developed for the treatment of COPD. Here we have optimized our engineering protocol by minimizing CXCL8 mutations while conserving its *in vitro* dominant-negative activities. This novel CXCL8-based decoy (mtCXCL8) was further fused to human serum albumin (HSA) to overcome the typically very short serum half-life of chemokine-based biologics. We are therefore able to present here an entirely novel CXCL8-based biologic (hsa/mtCXCL8) which reflects our threefold modification strategy – increasing GAG-binding affinity by minimal mutagenesis, GPCR knockout, and fusion to HSA – thus representing a comprehensive and novel approach towards addressing chronic CXCL8-driven diseases.

**Results:**

In the current study, we have investigated the immunomodulatory potential of our new decoy in a 3-D cellular tumor model (“BioMAP”) which relates the biomarker interaction profile of immune and tumor cells to a data-base mirrored biomarker read-out. The obtained BioMAP results suggest an impact of hsa/mtCXCL8 on the immune compartment of the VascHT29 cell model by modulating cytokine levels and inhibiting immune cell activation markers. When combined with Keytruda (Pembrolizumab), a PD-1 inhibitor, it enhances some of its known activities, indicating potential synergistic effects, but further investigation is needed due to the observed increase in soluble IL-6 and limitations in dose selection for future *in vivo* studies.

**Discussion:**

By prolonging the presence of engineered chemokine mutants in the bloodstream and optimizing their stability, these strategies aim to enhance the therapeutic efficacy of CXCL8-based interventions, offering promising avenues for the treatment of several CXCL8-mediated pathologies, including cancer.

## 1 Introduction

CXCL8 belongs to the class of inflammatory chemokines, which is expressed by monocytes, T-cells, neutrophils, natural killer cells, endothelial cells, fibroblasts and epithelial cells. Their main function is the mediation of leukocyte trafficking (1). CXCL8 exerts its function by activating two G-protein coupled receptors (GPCR) chemokine receptors, CXCR1 and CXCR2. Upon binding the receptor is internalized, recycled and recurs on the cell surface (2, 3). Chemokine signaling leads to the activation of leukocytes, which can direct cell migration to sites of inflammation along a chemokine gradient. CXCL8 thus plays a role in chronic obstructive pulmonary disease (COPD), neutrophilic asthma, cystic fibrosis, rheumatoid arthritis and other autoimmune diseases (4), and, moreover, in cancer.

CXCR2 serves as a G-protein-coupled receptor for human CXC chemokines, including CXCL1, CXCL2, CXCL3, CXCL5, CXCL6, CXCL7, and CXCL8 (5–7). CXCR2 is overexpressed in several cancer types, including breast, prostate, colorectal, and lung cancers (6). Its upregulation is often associated with poor prognosis and increased metastatic potential. Several studies have explored CXCR2 antagonists in therapeutic strategies for cancer (8, 9). CXCR2 exerts its pro-metastatic effects through various mechanisms, including the promotion of cancer cell migration, invasion, and angiogenesis (10). CXCL8, a known CXCR2 activator, also interacts with multiple intracellular signaling pathways to produce coordinated effects. Neovascularization, a basis for promoting tumor growth and metastasis, is now seen as a critical function of CXCL8 in the tumor microenvironment (TME) (8). Primarily known for regulating neutrophil migration and recruitment to sites of inflammation, CXCR2 also influences the migration of immune-suppressive myeloid-derived suppressor cells (MDSCs) into the tumor microenvironment and the pre-metastatic niche (11). Previous research has highlighted the widespread expression of CXCR2 not only in tumor cells but also in various immune cells including granulocytes, monocytes, mast cells, and some natural killer cells, as well as in endothelial and myeloid cells, contributing to tumor growth and vasculature development (12). Elevated levels of CXCR1 or CXCR2 have been associated with increased melanoma cell proliferation and invasion, whereas inhibition of these receptors has been shown to impede melanoma cell growth, movement, and vascularization both in laboratory settings and animal models (13, 14). Additionally, heightened expression of CXCR2 has been correlated with poorer prognosis and survival rates. Heightened levels of CXCL8 along with overexpression of CXCR1 or CXCR2 have been implicated in promoting tumor cell proliferation and metastasis through the activation of PI3K/AKT and ERK1/2 MAPK signaling pathways (8, 13, 15, 16). In a mouse model study, inhibition of the CXCL8-CXCR1/2 pathway using CXCL8-neutralizing antibodies resulted in a significant reduction in tumor growth, angiogenesis, and metastasis (17). A neutralizing antibody targeting CXCR2 exhibited a more pronounced suppressive effect on the migration and invasion of esophageal squamous cell carcinomas cell lines stimulated by tumor-associated macrophages -like peripheral blood monocytes -derived macrophages, compared to the neutralizing antibody against CXCR1 and CXCL8 (14).

In the course of immune cell mobilization, chemokines not only interact with GPCRs but also with Glycosaminoglycans (GAG). GAGs are polyanionic linear polysaccharides consisting of repeating disaccharide units (18). The hexosamines D-glucosamine or D-galactosamine are connected via a glycosidic linkage to D-glucuronic or L-iduronic acid (19). GAGs are categorized into four distinct groups based on their fundamental structural features and glycosidic linkages: (1) heparan sulfate/heparin (HS/Hep), (2) chondroitin sulfate/dermatan sulfate (CS/DS), (3) hyaluronan (HA), and (4) keratan sulfate (KS) (20, 21). GAGs are covalently attached to a core protein, forming the so-called proteoglycan. Proteoglycans (PGs) play a pivotal role in intercellular communication, primarily owing to their strategic positioning on the cell surface and within the extracellular matrix (ECM). They dynamically govern essential cellular processes such as adhesion, migration, proliferation, and differentiation (22–25).

The localization of proteoglycans in either the cell membrane or the external matrix is determined by the protein component, whereas the GAG component facilitates diverse interactions with extracellular ligands, encompassing growth factors, chemokines, and adhesion molecules (26–28). The inherent structural flexibility of GAGs allows for intricate modifications, introducing notable complexity and variability. For instance, N- and O-sulfation imparts a considerable negative charge to GAGs (29). *In vivo*, the dynamic interplay between chemokines and their receptors transpires within the microvasculature of tissues, necessitating the creation of a localized concentration gradient (30). This gradient is facilitated through the interaction of chemokines with cell surface GAGs. Moreover, GAGs serve a protective function by shielding chemokines from degradation and sequestration, presenting them prominently on the surface of the homing tissue. This presentation is imperative for the *in vivo* efficacy of specific chemokines (31–33). Binding between chemokines and GAGs is mediated mainly through electrostatic forces between the highly negative sugar and the basic regions of the chemokine, but also hydrogen bonding and Van der Waals interactions contribute to this tight interaction (9, 20). GAGs and PGs are essential components of the extracellular matrix (ECM), a complex network of molecules that provides structural support and regulates cell behavior in tissues (34). In cancer, alterations in the composition and organization of the ECM, including changes in GAGs and proteoglycans, play critical roles in tumor progression and metastasis (35). These alterations, including increased stiffness, allow cancer cells to sense mechanical cues and adapt to their environment (36). GAGs engage with a multitude of signaling molecules, exerting multifaceted influences on cancer at various levels and they are able to affect the physical properties of the ECM (37). Aberrations in glycosaminoglycan levels and composition, including alterations in sulfation patterns and molecular weight, are present in diverse cancer types and are linked to a modulated affinity for their binding molecules. Diverse sulfation patterns have been demonstrated to regulate the biological functions of glycosaminoglycans and their specificity in binding to growth factors, extracellular matrix (ECM) proteins, chemokines, and cytokines in various cell types and tissues (38). Transmembrane PGs, like syndecan, mediate cellular responses to these mechanical stresses by activating MAPK signaling, promoting cell survival and migration (39). GAGs also contribute to ECM stiffening by regulating collagen structures, thus influencing tumor growth. Dysregulated interactions between PGs/GAGs and other molecules in the tumor microenvironment (TME) contribute to ECM remodeling (35, 40).

Particularly during conditions of blood flow at the endothelial surface, the capacity of chemokines to bind with GAGs assumes a pivotal role in sustaining a chemotactic gradient at the cellular surface or within the extracellular matrix (41). Dilution of the chemokine into the bloodstream or tissue fluids poses a significant risk of rapid loss of biological functionality. Additionally, the interaction between GAGs and chemokines may serve a dual purpose, not only preventing protein unfolding but also contributing substantially to chemokine stability. Consequently, this protective interplay with GAGs may extend the duration of chemokine activity, safeguarding it from proteolytic degradation in the *in vivo* milieu. This axis is involved in numerous physiological and pathophysiological processes, such as hemostasis, growth factor control, anticoagulation, inflammation and pathogen attachment (21, 42).

Due to the involvement of Leukocyte trafficking in inflammatory and autoimmune diseases, several approaches were deployed to target this chemokine network. To interfere with chemokine signaling, we have generated dominant-negative decoy chemokines which exhibit increased GAG binding affinity combined with a knock-out of the GPCR-binding domain (4). More specifically, the pro-inflammatory activity of CXCL8 was disabled by deleting the first six amino acids (containing the ELR motif) of the sequence, which are essential for CXCR1/2 activation. On the other hand, exchanging water-exposed amino acids in the GAG binding domain (i.e. the C-terminal helix of CXCL8) to basic amino acids, the affinity of the chemokine for GAGs was significantly increased. Such decoy chemokines were shown to displace the corresponding wildtype protein from its GAG ligand (and prevent anew binding) – the dominant feature of the decoy – while being inactive with respect to its corresponding GPCR(s) – the negative feature of the decoy. By site-directed mutagenesis of the chemokine, we tried to enhance electrostatic forces between chemokine and GAGs, whereas hydrogen bonding and Van der Waals interactions were designed to remain intact. This is important since our CXCL8 decoy mutant protein(s) are not supposed to bind to GAG ligandss, other than its potential cognate GAG motif expressed on CXCL8-targeted cells. This proposed ligand binding specificity is supposed to be driven, like in protein-DNA/RNA interactions, mainly by hydrogen bonding and Van der Waals interactions, whereas affinity is dominated by electrostatic forces. Because our chemokine mutant design is based on maintaining a potentially buildt-in GAG-binding specificity (with respect to the wild type chemokine), we do not expect adverse reactions due to downstream signaling via GAGs on chemokine target cells.

Our chemokine mutant proteins were initially designed using computational energy minimization and molecular dynamic simulation. Experimentally the dominant-negative characteristics of mutants like mtCXCL8 (which is CXCL8[Δ6 E70K N71K]) were investigated by isothermal fluorescence titrations or surface plasmon resonance for increased GAG binding affinity, and by neutrophil mobilization/migration experiments in the modified Boyden chamber for knocked-out GPCR activity (43).

Due to their small size chemokines, the serum half-life of chemokines is quite short and the renal clearance occurs too fast. Since daily administration of a decoy protein may pose issues of treatment compliance or the need for specialized personnel for the administration, in the case of intravenous injection, we have investigated several fusions of decoys to human serum albumin (HSA). HSA serves as a multifaceted carrier in the bloodstream, playing a crucial role in transporting various molecules and maintaining physiological homeostasis (44). In an earlier study, we were able to engineer an HSA-CCL2 mutant fusion protein, which showed increased half-live compared to the unfused decoy protein. This chimera did not lead to a loss in GAG binding affinity but showed increased stability and bioavailability (45, 46).

The development of a mutant derived from CXCL8, characterized by an augmented affinity for GAGs, represents a promising avenue in the therapeutic intervention for chronic inflammatory diseases and cancer. The efficacy of CXCL8 decoys has been demonstrated in various mouse models (24, 25). However, a possible limitation of its application for chronic treatments is the fact that the substance necessitates regular administration owing to its short half-life (46–48). The fusion of a CXCL8 mutant with HSA presents an innovative strategy to prolong its presence in the bloodstream by preventing early renal secretion. The conjugation with HSA not only enhances the stability of the mutant but also extends its serum half-life, thereby optimizing its therapeutic efficacy. Structural stability of the mutant CXCL8 domain is due to the scaffolding effect of HSA which relates to its function as natural serum protein carrier without affecting the structure-function of the cargo proteins.

Myeloid-derived suppressor cells (MDSCs), which are not present in healthy individuals, expand in tumor-bearing hosts and contribute to tumor immune evasion through various mechanisms, including oxidative stress and nutrient depletion via inducible nitric oxide synthase (iNOS) and arginase, transforming growth factor-beta (TGFβ), IL-10, cyclooxygenase-2 (COX2), and indoleamine 2,3-dioxygenase (IDO) production (49, 50). MDSCs exert potent suppression of CD8+ T-cell activity by employing the expression of Fas-ligand, which engages with Fas receptors on tumor-infiltrating lymphocytes (TILs), triggering the apoptotic demise of these critical immune cells and they are also involved in the recruitment of tumor-associated macrophages (11). Tumor-infiltrating leukocytes, type 2 macrophages and MDSCs are primarily recruited by cancer cells to establish a microenvironment conducive to tumor growth, aiming to suppress the anti-tumor immune response (51). Inhibiting MDSC trafficking to the tumor enhances the potency of PD-1 (programmed cell death protein 1) checkpoint blockade (49, 50). The upregulation of PD-1 represents a pivotal mechanism driving the exhaustion of effector T-cells, fostering an environment conducive to tumor immune evasion. Through the interaction between PD-1 on T-cells and PD-L1 on neoplastic cells, tumors effectively evade anti-tumor immunity. The blockade of PD-1 stands as a cornerstone in cancer immunotherapy, with Pembrolizumab (Keytruda), a potent humanized monoclonal anti-PD-1 antibody, undergoing rigorous exploration across a broad spectrum of malignancies (52, 53). Antibodies directed against the receptor or the ligand can loosen the immune regulatory brakes, enabling the T-cell to become effective again and overcome tumor immune subversion (53).

Thus, CXCR2 is identified as a novel target for therapies aiming to inhibit MDSC recruitment and provides a rationale for combining immune checkpoint inhibitors with agents designed to prevent MDSC-mediated immune suppression in cancer therapy (49, 54). Understanding the intricate roles of CXCR2 in both tumor immune evasion and metastasis offers promising avenues for developing more effective cancer treatments. In a murine model of prostate cancer, the introduction of a CXCR2 antagonist or the infusion of bone marrow-derived CXCR2 Knockout macrophages resulted in a striking decrease in tumor growth. Furthermore, this intervention prompted a profound reprogramming of tumor-associated macrophages (TAMs) toward a highly beneficial pro-inflammatory M1 phenotype (55).

Given their pivotal role in the tumor microenvironment (TME), directing interventions towards GAGs and leveraging their interactions with critical immune mediators like chemokines and immune receptors represents intriguing anticancer approaches. Therefore, we explored the antitumor potency of our fusion construct in a BioMAP assay (DiscoverX) in combination with pembrolizumab (Keytruda). Our HSA-CXCL8 fusion construct is thought to displace the naturally occurring CXCL8 from the surface of GAGs present and often overexpressed in the tumor and thus blocking peripheral blood mononuclear cell (PBMC) influx, which is studied in combination with a marketed PD-1 antibody that circumvents the T-cell blockade and reinstalls T-cell function. The inhibition of MDSC trafficking to the tumor is thought to enhance the potency of PD-1 checkpoint blockade. Blocking this CXCL8/CXCR2/GAG axis is a novel therapeutic strategy that could potentially reverse the tumor immune evasion.

## 2 Materials and methods

### 2.1 Materials

Low molecular weight (LMW) heparin, Heparan sulfate and GAG binding plates were purchased from Iduron (Manchester UK). All chemicals unless stated otherwise were obtained from Merck (Darmstadt, Germany). mtCXCL8, CXCL8 and hsa/mtCXCL8 were produced in house. Phosphate buffered saline (PBS) contains 10 mM phosphate buffer and 137 mM NaCl with a pH of 7.2.

### 2.2 Expression and purification

#### 2.2.1 Protein expression and purification of dnCXCL8 and CXCL8

Expression plasmids for mtCXCL8 (= CXCL8[Δ6 E70K N71K]) and wildtype CXCL8 were purchased from ATUM in the pJExpress411 expression vector. Vectors were transformed into BL21 (DE3) Star E.coli cells (Invitrogen, Carlsbad, CA, USA) and plated on LB plates containing 30 µg/mL Kanamycin. Overnight cultures were prepared using a single colony and main cultures were grown in 3-liter Erlenmeyer flasks under 200 rpm shaking at 37°C in LB broth containing 30 μg/mL Kanamycin. At an OD600 of 0.8 protein production was induced with 0.5 mM isopropyl β-D-thiogalactopyranoside (IPTG). After 3 hours E.coli cells were harvested by centrifugation for 15 minutes at 6000g and purification was performed as described before (4).

#### 2.2.2 Protein expression and purification of hsa/mtCXCL8

The expression of hsa/mtCXCL8 (which is a genetical fusion protein consisting of human serum albumin, HSA, linked C-terminally by the flexible peptide CCCCS to the N-terminus of mtCXCL8, see below for the amino acid sequence of the fusion construct) was conducted utilizing Pichia pastoris as the expression host in a 1-liter Multifors bioreactor (Infors AG, Bottmingen, Switzerland). The two-step fermentation process encompassed a growth phase on glycerol followed by a production phase on methanol as the sole carbon source. Pichia pastoris CBS7435 muts-PDI strain was inoculated in a starting volume of 0.4 liters of fermentation medium (1/2 BSM containing 40 g/l glycerol) at a temperature of 28°C and pH 5.0. The temperature was gradually decreased from 28 to 24°C, and the pH was raised to 6.0 during the last 2 hours of the glycerol-fed batch and maintained at that level throughout the production period.

Amino acid sequence of hsa/mtCXCL8: DAHKSEVAHRFKDLGEENFKALVLIAFAQYLQQAPFEDHVKLVNEVTEFAKTCVADESAENCDKSLHTLFGDKLCTVATLRETYGEMADCCAKQEPERNECFLQHKDDNPNLPRLVRPEVDVMCTAFHDNEETFLKKYLYEIARRHPYFYAPELLFFAKRYKAAFTECCQAADKAACLLPKLDELRDEGKASSAKQRLKCASLQKFGERAFKAWAVARLSQRFPKAEFAEVSKLVTDLTKVHTECCHGDLLECADDRADLAKYICENQDSISSKLKECCEKPLLEKSHCIAEVENDEMPADLPSLAADFVESKDVCKNYAEAKDVFLGMFLYEYARRHPDYSVVLLLRLAKTYETTLEKCCAAADPHECYAKVFDEFKPLVEEPQNLIKQNCELFEQLGEYKFQNALLVRYTKKVPQVSTPTLVEVSRNLGKVGSKCCKHPEAKRMPCAEDYLSVVLNQLCVLHEKTPVSDRVTKCCTESLVNRRPCFSALEVDETYVPKEFNAETFTFHADICTLSEKERQIKKQTALVELVKHKPKATKEQLKAVMDDFAAFVEKCCKADDKETCFAEEGKKLVAASQAALGLGGGGSCQCIKTYSKPFHPKFIKELRVIESGPHCANTEIIVKLSDGRELCLDPKENWVQRVVEKFLKRAKKS.

For further purification of the full-length hsa/mtCXCL8, a two-step purification process was employed. In the initial step, a strong cation exchange resin, Fractogel EMD SO_3_^–^ (Merck, Darmstadt, Germany), specifically interacting with the chemokine portion of the construct, was utilized. The second step involved an affinity chromatography resin, Blue Sepharose 6 Fast Flow (Cytiva, Marlborough, MA, USA), where Cibacron™ Blue 3G is covalently attached to the Sepharose 6 Fast Flow matrix via the triazine coupling method. This blue dye binds numerous proteins, including albumin, interferon, lipoproteins, blood coagulation factors, and several enzymes such as kinases and dehydrogenases.

For the initial purification, the supernatant obtained from P. pastoris fermentation was diluted 1:2 using a 50 mM Tris pH 8 buffer (low salt buffer) and loaded onto Fractogel EMD SO_3_^–^ pre-equilibrated in low salt buffer. Elution was achieved by applying a linear gradient from 0 to 2M NaCl in 50 mM Tris pH 8 over 10 column volumes (CV). The protein-containing fractions were pooled and diluted 1:12 in a low salt buffer for the second purification step. The diluted protein solution was loaded onto Blue Sepharose 6 Fast Flow pre-equilibrated in 50 mM Tris pH 8. Elution followed the same procedure as the first step. Protein concentration was increased through ultrafiltration using Amicon Ultra-15 (Ultracel-3k, Millipore, Merck), and buffer exchange was performed by dialysis against PBS. A third purification step was introduced to enhance the binding affinity of hsa/mtCXCL8, using the cation exchange resin SP Sepharose Fast Flow (Cytiva) with the same buffers as mentioned earlier. Elution, buffer exchange to PBS, and concentration was determined by UV280 measurement.

### 2.3 Isothermal fluorescence titration

The interaction of mtCXCL8, CXCL8 and hsa/mtCXCL8 with GAGs has been studied using IFT. Binding affinities can be measured via this method with high sensitivity and robustness by monitoring a decrease in fluorescence signal. This quenching arises from structural re-arrangement of the protein post-interaction with the ligand; with higher binding affinity correlating to increased quenching of the intrinsic tryptophan residue. IFT measurements were conducted on a Jasco FP6500 Spectrofluorometer, employing an excitation wavelength of 280 nm over the range of 300–400 nm. Slits were set to 3 nm for excitation and 5 nm for emission. Titrations were performed with Heparin and Heparan Sulfate from Iduron, with additions between 50 nM to 1000 nM of ligand. The protein was preequilibrated for 30 minutes in the fluorometer prior to ligand addition. After each GAG addition, the solution was further equilibrated for 1 minute before measuring its spectrum.

Samples were measured in triplicates and the binding isotherms were generated by plotting the relative change in fluorescence intensity (ΔF/F_0_I) against the concentration (C) of added ligand. The data were analyzed using Origin 8.0 through non-linear regression fit, considering a fixed number of binding sites due to challenges in estimating specific binding sites and an incomplete understanding of oligomerization behavior (55).

### 2.4 Surface plasmon resonance

All measurements were conducted on a Biacore X100 system (Cytiva) at a constant temperature of 25°C. The running buffer consisted of PBS with 0.005% Tween (Merck). C1 sensor chips (Cytiva) were prepared according to the manufacturer’s instructions, involving activation of C1 carboxyl groups with EDC/NHS, coating with neutravidin (0.2 mg/mL in acetate buffer pH 4), and subsequent blocking with ethanolamine. Flow runs were set at 5 μL/min with a contact time of 10 minutes for all reagents. Immobilization of biotinylated GAGs (heparin, heparan sulfate) on the C1 chip surface was performed at a flow rate of 5 μL/min and a concentration of 20 μg/mL for heparin and 1 µg/ml for HS for 55 seconds.

The reference cell was exclusively immobilized with neutravidin, acting as a control to measure background chemokine binding. Each binding measurement involved testing seven different concentrations of the respective chemokine in quadruplicate, with the second-lowest concentration injected twice as a control. Contact times for all injections and dissociations were set at 120 seconds at 30 μL/min over both flow cells. A regeneration solution (1 M NaCl) was introduced directly after each dissociation time at 30 μL/min with a 60-second contact time per cycle. Data analysis was performed using Biacore Evaluation software 1.0, and affinity constants were determined through a simple 1:1 equilibrium binding model, plotting Req against the analyte concentration. The steady-state formula corresponding to the Langmuir adsorption equation, provided by the Biacore Evaluation Software, was used for fitting (55).

### 2.5 ELISA-like competition

#### 2.5.1 Biotinylation of chemokines

Before biotinylating the chemokine, a buffer exchange to 0.1 M MES was carried out using Amicon Ultracel 3K 4 mL (Millipore, Merck, Darmstadt, Germany) to establish optimal reaction conditions. Subsequently, the protein underwent incubation with a 20-molar excess of EZ Link Penthylamine Biotin (Thermo Scientific, Waltham, MA, USA) and a 10-molar excess of EDC (Thermo Scientific) for 2 hours at room temperature with gentle agitation. Desalting was then conducted using ZEBA desalting columns (Thermo Scientific) following the manufacturer’s protocol. The protein concentration was assessed through photometric measurements.

#### 2.5.2 ELICO protocol

2,5 µg of GAGs (per well) were diluted in 1x PBST and coated onto Iduron plates (Iduron), with the preparation process carried out overnight at room temperature. Subsequent to a precise washing step executed by an automatic platewasher (Tecan, Männedorf, Switzerland), the pre-coated GAG plates underwent a 1-hour incubation at room temperature with 250 nM biotinylated CXCL8. Following another washing step to eliminate any unbound biotinylated CXCL8, the plates were subjected to a 2-hour incubation with various competitor concentrations, spanning from 50 μM to 3 nM, all diluted in PBS and each concentration measured 5-times. This phase, executed at room temperature, aimed to displace the bound biotinylated CXCL8. Employing an ELISA-like setup, the detection of the remaining biotinylated CXCL8 was facilitated. After an additional thorough washing step, the plates were subjected to an hour-long incubation at room temperature with high-sensitivity Streptavidin HRP (Thermo Fisher), diluted in 0.2% dry milk. This agent binds specifically to the non-displaced biotinylated CXCL8 on the plate. Following the removal of unbound Streptavidin, the analysis of the plate was conducted by introducing the substrate Tetramethylbenzidine (TMB, Merck), resulting in a distinct blue color change. Upon halting the reaction with sulfuric acid, the absorbance at 450 nm was quantified using a Beckman Coulter DTX 800 Multimode Detector (Vienna, Austria), with correction at 620 nm. The reference (OD620) values were subtracted from the sample values (OD450), and the Mean and Standard Deviation of the replicates were calculated. A thorough data analysis was carried out using specialized statistical software Origin^®^ (OriginLab Corporation, Northhampton, MA, USA) and IC50 values were calculated.

### 2.6 Neutrophil chemotaxis

The chemotactic activity of mtCXCL8 and hsa/mtCXCL8 was compared to unmodified wildtype CXCL8 using a 48-well Micro Chemotaxis Chamber (Neuroprobe, Gaithersburg, MD, USA) with a 5 μm pore size PVPfree polycarbonate membrane (Neuroprobe). Human whole blood was obtained from healthy volunteers by venipuncture into heparinized tubes (Vacuette, Greiner BioOne, Kremsmünster, Austria). Neutrophils were isolated using Ficoll-Paque™ PLUS (Cytiva). Plasma and the Ficoll fraction were aspirated down to the red blood cell pellet and these pellets were resuspended in up to twice the original blood volume with HBSS without calcium and magnesium (-/-, Thermo Fisher). Red blood cells were mixed with dextran sulfate solution (Merck) to achieve a final concentration of 1%. Cells settled for 1 hour at RT without disturbing. The remaining red blood cells were lysed with ice-cold water and Neutrophils were washed several times with HBSS -/- (Thermo Fisher).

After washing cells were resuspended in HBSS with calcium and magnesium (+/+, Thermo Fisher) to a final concentration of 3,5x 10^6^ cells/mL. Protein dilutions ranging from 15-1500 nM in HBSS +/+ were placed in the lower chambers, each concentration measured in triplicates on three independent chemotaxis chambers, including buffer-only controls. 50 µL of freshly prepared neutrophil cell suspension in HBSS +/+ were seeded in the upper chamber and incubated for 30 minutes at 37°C and 5% CO_2_. After incubation the filter was removed from the chamber, non-migrated cells were washed away and cells attached to the lower side were fixed with methanol and stained with Hemacolor solutions (Merck, Darmstadt, Germany). Cells were then counted at 400x magnification in 5 randomly selected microscopic fields per well and the chemotactic indices were calculated by dividing the mean migrated cells through the cells that were observed in the background. Finally, the mean and standard deviation of the triplicate measurement of 3 independent experiments were plotted, after background correction, against the chemokine concentration.

### 2.7 Transwell

The inhibited migratory response by induction of different substances was tested in a transwell assay. 35 000 EA.hy926 (ATCC^®^ CRL-2922) cells per well were seeded on collagen coated 96-well Transwell plates (HTS Transwell, Corning) and grown for 48 hours at 37°C and 5%CO2. Cells were stimulated for 4 hours with 50 ng/mL TNFα. For the transmigration 200 000 cells were necessary per well. Therefore 1x 10^7^ Stimulated Neutrophils were harvested, diluted in 10 mL HBSS -/- and labelled with 10 µL of 2 mM Calcein AM for 30 minutes at 37°C and 5% CO_2_. After labelling cells were washed twice with HBSS -/- and pellet was resuspended in 4 mL 20mM HEPES in HBSS +/+. Dilutions of 20 nM wT chemokines were pipetted into the lower wells. All samples were measured in triplicates. 40 µL of cells suspension was mixed with 40 µl of the appropriate inhibitory agents and added to the upper wells. Transmigration lasted for 2 hours at 37°C and 5% CO_2_. Detection was performed by measuring the fluorescence intensity of migrated cells in the lower wells at 495 nm (excitation)/515 nm (emission) using the SpectraMax M3 Plate Reader (Molecular Devices, CA, USA). Statistical analyses were performed with GraphPad v5.04 using student’s t-test.

### 2.8 Circular dichroism spectroscopy

Circular dichroism (CD) measurements of 10 μM of the hsa/mtCXCL8, CXCL8 and mtCXCL8 in PBS were performed on a Jasco J-710 Spectropolarimeter (Easton,MD, USA). Data collection was performed between 190 and 250 nm with a response time of 1 s, a data point resolution of 0,2 nm, a scan speed of 50 nm/min, with a bandwidth of 1 nm and a sensitivity set to 100 mdeg. Each sample was measured 10 times and the mean spectrum of these repeats was analyzed. Cuvettes with 0.1 cm path length were used and five scans were averaged and background corrected in order to obtain smooth spectra. Mean residue ellipticities of the corrected spectra were calculated and plotted against the wavelength.

### 2.9 Chaotropic unfolding

Protein solutions at a concentration of 0.7 μM were prepared with varying guanidine concentrations ranging from 0 to 6 M (Thermo Fisher) and measured in triplicates. These solutions were then equilibrated for 30 minutes at room temperature (°C). Fluorescence scans, as described before, were recorded, and the wavelength shift of the protein with increasing guanidine concentration was analyzed using the Boltzmann equation:


$$y=\text{A}2+\frac{\left(\text{A}1-\text{A}2\right)}{1+\text{exp}\left(\frac{x-x0}{dx}\right)}$$

Here A1 represents the initial wavelength, A2 is the final wavelength, and the fitted parameters include dx and x0.

### 2.10 DiscoverX BioMAP ^®^oncology VascHT29 combo ELECT

To assess the *in vitro* potentials of hsa/mtCXCL8 we have used the DiscoverX BioMAP^®^ (Eurofins Scientific, Fremont, CA, USA).

This standardised *in vitro* system allows to assess the effects of new therapeutics in development, utilising a complex cell-based assay, comparing the obtained data with a proprietary reference database with responses of >4000 test agents, and finally the use of proprietary informatic tools, to evaluate the mechanism, and initially indicate selectivity and safety.

We have selected the oncology VascHT29 combo ELECT system, which comprises primary venular endothelial cells, HT-29 colorectal adenocarcinoma cell line both stimulated for 48 hrs via the T cell receptor Sag and human peripheral blood mononuclear cells. These cells together mimic the tumor-host microenvironment and capture the interactions between tumor cells, the host vascular network and the infiltrating immune cells associated with angiogenesis.

Biomarker readouts in the supernatants are measured using multiplex and include markers of immune-related (e.g. IL-6, IL-10, IL-2), inflammation-related (e.g. CCL-2, CXCL-10, VCAM-1 sINFγ), angiogenesis-related (uPAR) and tumor-related activities, all of them listed in the X axes.

After incubation with different concentrations of the has/mtCXCL8 or its vehicle, the relative expression levels of the biomarkers are analysed.

As reported by the service provider, the biomarker activities are annotated when values are significantly different from vehicle controls (p<0.01), are outside the so-called significant envelope and have an effect size of > 20% (|log10 ratio| > 0.1) versus the vehicle control.

In the combination studies, the biomarker activities are annotated when values are significantly different from both individually tested agents (p<0.01) and when at least one of the tested agents has an effect size of > 20% (|log10 ratio| > 0.1) versus the vehicle control.

We have initially tested hsa/mtCXCL8 at concentrations of 25; 500; 10,000 and 200,000 ng/mL. Based on the results of the dose response, the highest dose was then tested in combination with Keytruda (Pembrolizumab), a humanized antibody that targets the programmed cell death protein 1 (PD-1) receptor avoiding the binding of both of its ligands (PD-L1 and PD-L2), to explore possible synergistic effects.

### 2.11 Pharmacokinetic assessment in mice

Animal care and handling procedures including providing of food and water ad libitum, 12-hour light and dark cycle and keeping in air-conditioned cages were performed in accordance with the European guidelines and all the experiments were conducted under conditions previously approved by the local animal ethics committee.

6 weeks old female Balb/CcAnNCrl mice (Charles River, MA, Wilmington, USA) were treated with subcutaneous or intravenous injections of hsa/mtCXCL8 (386 µg/kg body weight, corresponding to 40µg/kg of the un-conjugated protein) in the lateral tail vein. Group size was determined as n=3/time point. At defined time points blood was collected by heart puncture of deeply anesthetized mice. The concentration of hsa/mtCXCL8 in serum was determined using a human Serum ELISA kit (Abcam, Cambridge, UK). ELISA setup was performed according to the manufacturer’s instructions.

## 3 Results

### 3.1 Far-UV CD spectroscopy and chaotrope-induced unfolding

**Figure 1:**
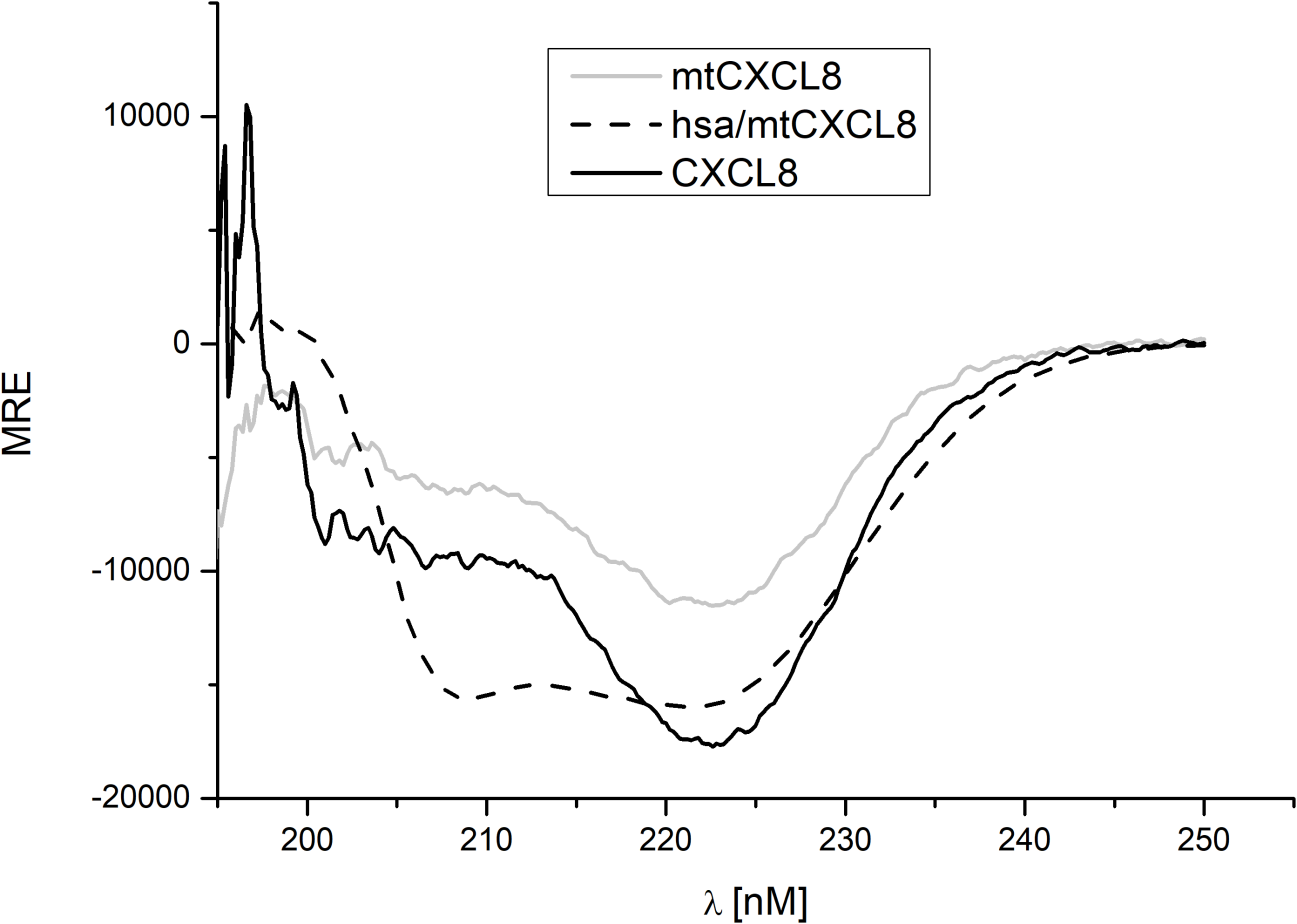
Far-UV CD measurement (mean residue ellipticity, MRE) of CXCL8, mtCXCL8 and hsa/mtCXCL8. Spectra were background corrected and five scans were recorded to obtain a smooth spectrum.

For every newly engineered protein construct it is important to evaluate their structural composition and conformational stability, especially if they are designed for potential therapeutic use in humans. Far UV Circular Dichroism (CD) Spectroscopy is a powerful analytical technique employed in biochemistry and structural biology particularly to investigate the secondary structure of proteins, peptides, and other biomolecules. This spectroscopic method utilizes circularly polarized light in the far-ultraviolet (UV) wavelength range, typically in the range of 190 to 250 nm, since this covers the CD resonance absorption energies of peptide bonds in distinctive secondary structural elements. As light passes through a chiral medium, such as a protein solution, the different absorption of left- and right-circularly polarized light results in a difference in the absorption intensity, known as circular dichroism. In the present study, we have investigated the secondary structure of hsa/mtCXCL8 in comparison to mtCXCL8 and CXCL8. The far-UV spectra of the proteins were recorded under quasi-physiological (PBS) conditions. **Figure 1** depicts the recorded spectra of our measurements, and the numerical analysis regarding secondary structural elements is summarized in **Table 1**. The secondary structures of native CXCL8 is characterized predominantly by β-sheet arrangements, consisting of six β-sheets and two α-helices within the dimeric chemokine form (which is reflected in the 3-D structure of the chemokine obtained by NMR and X-ray crystallography, see for example https://doi.org/10.2210/pdb1IL8/pdb). This is in accordance with our obtained results from BeStSel analysis where over 35% of the calculated secondary structure can be attributed to antiparallel elements and only around 5-10% account for the alpha-helical part. The CD spectrum of mtCXCL8 exhibits less signal intensity over the entire spectral range which is indicative of a less molded conformation compared to wildtype CXCL8. Since human serum albumin primarily consists of alpha helices, constituting the majority of its mass compared to the chemokine segment, this dominance is evident in the CD spectra of hsa/mtCXCL8. The analysis revealed that the predominant portion is helical (45%), with only a smaller region attributed to beta sheets (19,4%) (see **Table 1**).

**Table 1:** Conversion of the CD spectrum using BestSel (http://bestsel.elte.hu/), which provides information on the secondary structure content.

	CXCL8	mtCXCL8	hsa/mtCXCL8
**Helix**	**11,4**	**5,1**	**45**
(regular)	9,5	5,1	31,2
(distorted)	1,9	0	13,8
**Antiparallel**	**30**	**35,2**	**19,4**
(left-twisted)	8,2	7,4	0
(relaxed)	0	3,4	1,3
(right-twisted)	21,8	24,4	18,1
**Parallel**	**0**	**0**	**3,3**
**Turn**	**19,9**	**20,6**	**17,8**
**Others**	**38,7**	**39,1**	**14,4**

RMSD denotes the root mean square deviation, while NRMSD represents the normalized root mean square deviation. Calculations were done between 200-250 nm.

Helix: % alpha helical content; Antiparallel: % antiparallel betasheetcontent; Parallel: % parallel beta-sheet content; Turn: % loop/turn content; Others: % non-regular secondary structural elements.

**Figure 2:**
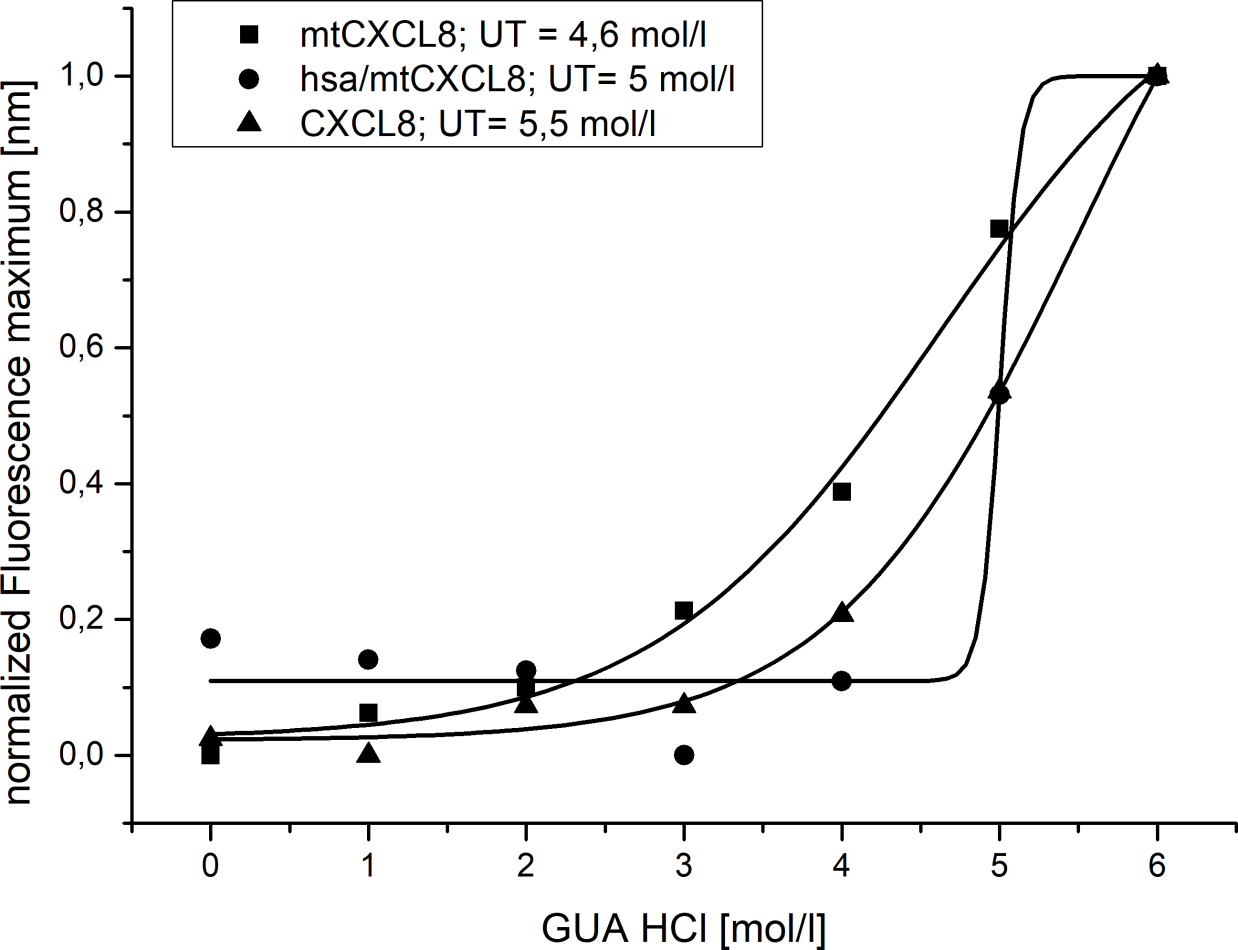
Normalized Guanidine induced Unfolding of CXCL8, mtCXCL8 and hsa/mtCXCL8 (n=3). Fluorescence wavelength shift was employed to track denaturation, with characterization based on the unfolding transition midpoint (UT).

Conformational stability was investigated using Guanidine-HCl- induced unfolding (see **Figure 2** and **Table 2**). The process of protein denaturation induced by guanidine involves the disruption of native protein structures due to the dismantling of non-covalent interactions, such as hydrogen bonds and hydrophobic forces. These interactions are essential for maintaining the protein’s original, functional conformation. Both CXCL8 and mtCXCL8 were found to be quite loosely folded in solution which is expressed in a flat unfolding curve which doesn’t show a clear transition point and consequently doesn’t reach a completely unfolded state up to a chaotrope concentration of 6M. Fusion to HSA apparently induced a significant structural stabilization of the CXCL8 mutant, which is expressed in a characteristic “sharp” sigmoidal unfolding curve with a “late” midpoint of transition at 5M Guanidine-HCl for hsa/mtCXCL8. This is indicative for a cooperative unfolding process. In chaotrope-induced unfolding experiments of proteins, cooperativity relates to the degree by which the denaturation unfolds as a synchronized and cooperative event (in contrast to a stepwise or irregular process). Cooperative unfolding suggests that various structural components of the protein, including secondary and tertiary structural elemengts, undergo simultaneous and coordinated changes in response to the denaturing agent. When a protein unfolds cooperatively, structural deflections in one part facilitates the unfolding of adjacent regions. This cooperative phenomenon results in a seamless and clearly defined transition from the folded to the unfolded state, typically expressed as a sigmoidal or S-shaped curve in denaturation profiles. Highly cooperative unfolding is frequently associated with well-folded and stable proteins, while non-cooperative unfolding may suggest structural heterogeneity or flexibility within the protein (56). The latter situation is apparently observed for CXCL8 and mtCXCL8.

**Table 2:** Calculation of the unfolding transition midpoint (UT, denoted as x0) and the relative cooperativity (dx).

	x0	dx
**CXCL8**	5,5	0,8
**mtCXCL8**	4,6	0,9
**hsa/mtCXCL8**	5	0,1

### 3.2 Glycosaminoglycan binding affinities

Isothermal fluorescence titration (IFT) is a powerful experimental technique used in biophysical studies to investigate the interaction between (intrinsically) fluorescent proteins and their ligands in solution. Experiments involve the stepwise addition of ligand (in our case GAGs), to a solution containing the protein. The protein fluorescence emission, particularly if due to intrinsic fluorophores like tryptophan, is sensitive to ligand-induced changes of its microenvironment. This is why ligand binding typically induces changes in fluorescence intensity. During an isothermal fluorescence titration, the fluorescence signal is recorded for each added ligand concentration, providing the concentration-dependent response profile of the bi-molecular interaction which is analyzed to extract the binding affinity (Kd value).

**Figure 3:**
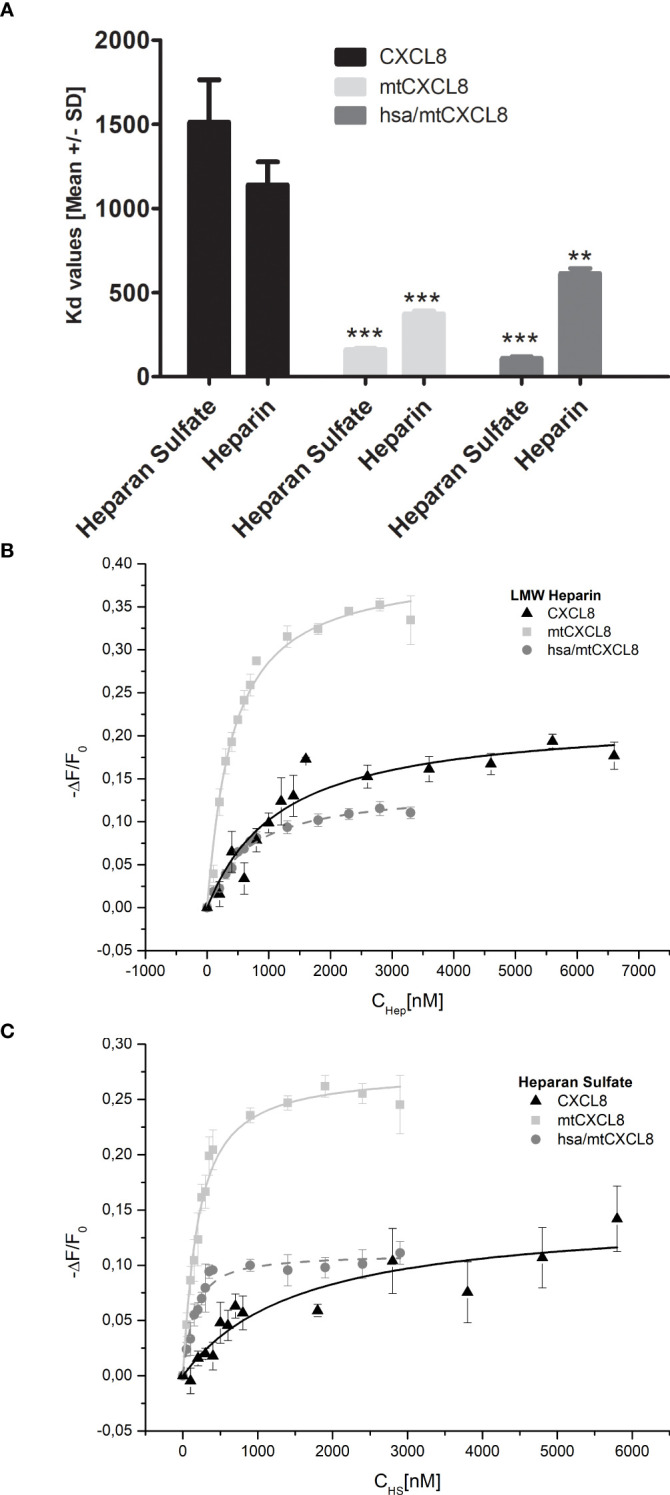
**(A)** Fluorescence binding isotherms for the interaction of CXCL8, mtCXCL8 and hsa/mtCXCL8 with **(B)** Heparin and **(C)** Heparan Sulfate. The fluctuations in fluorescence are depicted by plotting the normalized change in fluorescence (-ΔF/F₀, with F₀ representing the initial fluorescence intensity before the ligand addition) against the concentration of HS or LMW Heparin. These data are the average values from three separate experiments and were subjected to fitting procedures as detailed in the Method section. Student´s Ttest was performed comparing to their respective CXCL8 reference ** p < 0.01, *** p < 0.001 was considered statistically significant.

Using IFT, affinities (Kd values) for CXCL8, mtCXCL8 and hsa/mtCXCL8 binding to heparan sulfate and heparin were determined (see **Figures 3A–C**) mainly to find out the impact of coupling mtCXCL8 to HSA on GAG binding properties. Since mtCXCL8 itself was designed to have a higher affinity towards GAGs compared to wildtype CXCL8, these characteristics were found to be well conserved in hsa/mtCXCL8. Interestingly this increased affinity was more pronounced in binding to HS than in binding to heparin, although the latter is higher sulfated than HS. This selective discrimination for HS is indicative for specific GAG binding driven by interactions beyond purely electrostatic forces.

### 3.3 ELISA-like competition assay

Whereas we focused in IFT experiments on the one-to-one interaction of GAGs with chemokines and their mutants (i.e. bimolecular interactions), in the ELICO competition set-up we have investigated the potency of mtCXCL8 and hsa/mtCXCL8 to displace wildtype CXCL8 from HS (we focused here on this GAG since IFT experiments have shown that binding to heparin is less specific compared to HS, and because HS is the natural ligand of CXCL8 and not heparin). This set-up corresponds more to a naturally occurring situation in which the immunologically-active chemokines are bound to GAGs, which need to be displaced by our chemokine mutants in a potentially therapeutic setting. Therefore, in competition experiments the Kd values of the pre-bound wild type chemokine needs to be overcome by the Kd value of a chemokine mutant resulting in a IC50 value for the competitor. We have used an experimental set-up in which biotinylated CXCL8 is pre-incubated with heparan sulfate enabling a swift and reliable determination of IC50 values for a competing GAG-binding protein. CXCL8 was incubated with heparan sulfate and the decoys were added at increased concentrations. The detection of the remaining bound biotinylated CXCL8 was accomplished in an ELISA-like setting using Streptavidin coupled to HRP. The resulting signal, which changed in a concentration-dependent manner upon the addition of the competitor, was measured at 450 nm. This approach is representative of a tri-molecular event, in which the displacement potency of one protein is gauged by displacing another that is initially bound to the glycosaminoglycan (GAG) ligand on the plate surface. This setup more accurately mirrors the *in vivo* scenario, where chemokines are bound to GAG on endothelial surfaces, prompting the activation of immune cells to migrate toward sites of inflammation.

We have shown that CXCL8 biotinylation had only a minimal impact on GAG binding, resulting in no significant affinity difference between the Kd-value of wild-type CXCL8 (2.5 μM) and of the biotinylated protein (2.8 μM; see **Supplementary Figure 1**).

**Figure 4:**
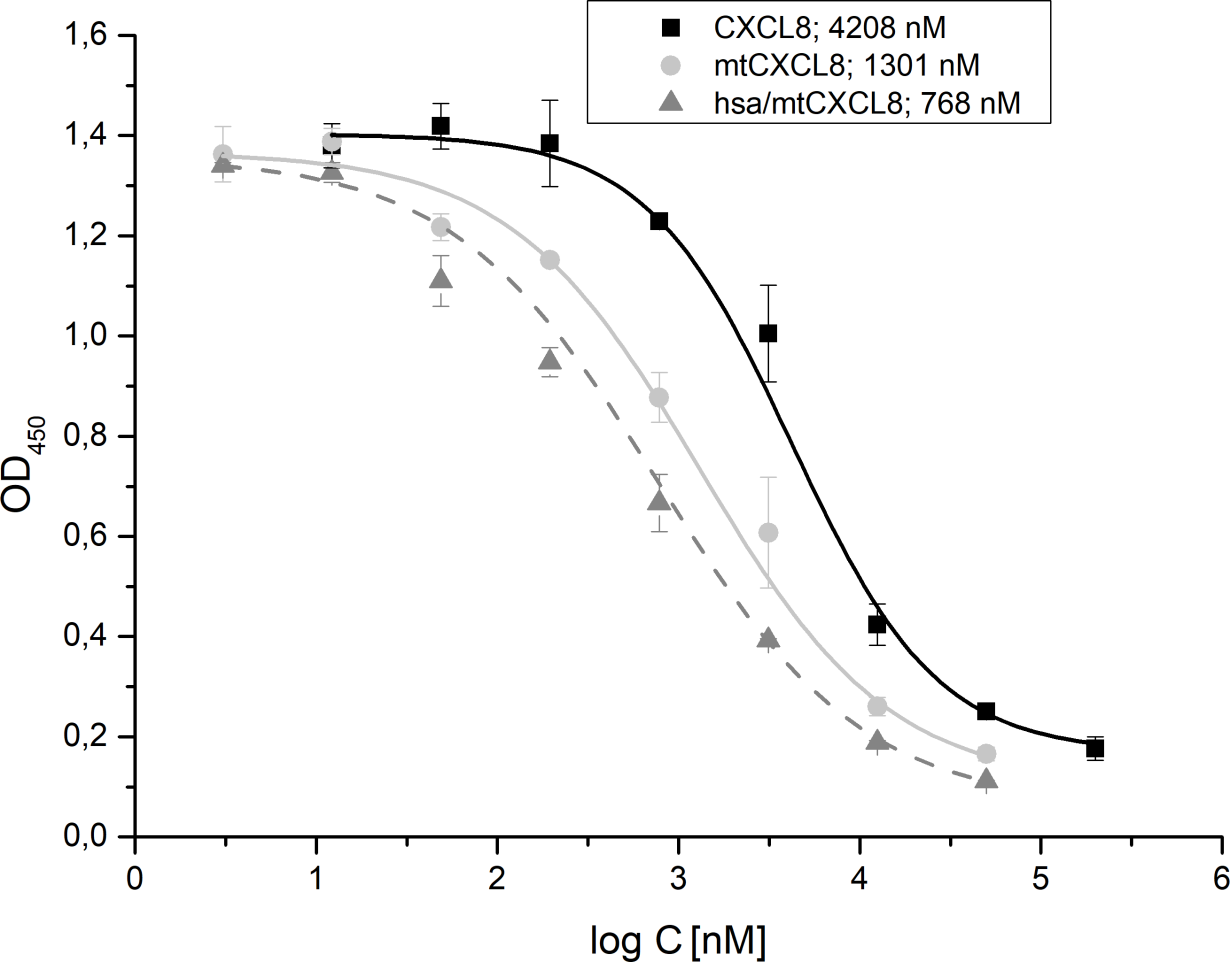
ELICO-derived displacement profile of biotinylated CXCL8 from HS by CXCL8, mtCXCL8 and hsa/mtCXCL8. IC50 values (n=5) were calculated for CXCL8 (4,21 ± 0,27µM ), mtCXCL8 (1,3 ± 0,24 µM) and hsa/mtCXCL8 (0,77 ± 0,16 µM).

The obtained competition data revealed that mtCXCL8 and hsa/mtCXCL8 exhibited IC50 values increased by 3-fold and 5,5-fold, respectively, relative to CXCL8 displacing CXCL8: IC50 (mtCXCL8) = 1,3 ± 0,24 µM, IC50 (hsa/mtCXCL8) = 0,77 ± 0,16 µM and IC50 (CXCL8) = 4,21 ± 0,27µM (**Figure 4**). These data show that the fusion construct is very potent in displacing its wildtype counterpart from the surface of GAGs.

### 3.4 Neutrophil mobilization and migration assay (Boyden chamber)

The Boyden chamber assay is a valuable technique in cell biology and immunology for studying cell migration, particularly the movement of immune cells through various barriers. It is a well-established and widely-used method to assess chemotaxis and cell mobilization/migration. It consists of a chamber with two compartments separated by a porous membrane. Cells are placed in the upper compartment, and a chemoattractant/chemokine is added to the lower compartment. The cells migrate through the membrane toward the chemoattractant, and the degree of migration can be quantified by staining and counting the cells that have migrated to the lower side of the membrane.

**Figure 5:**
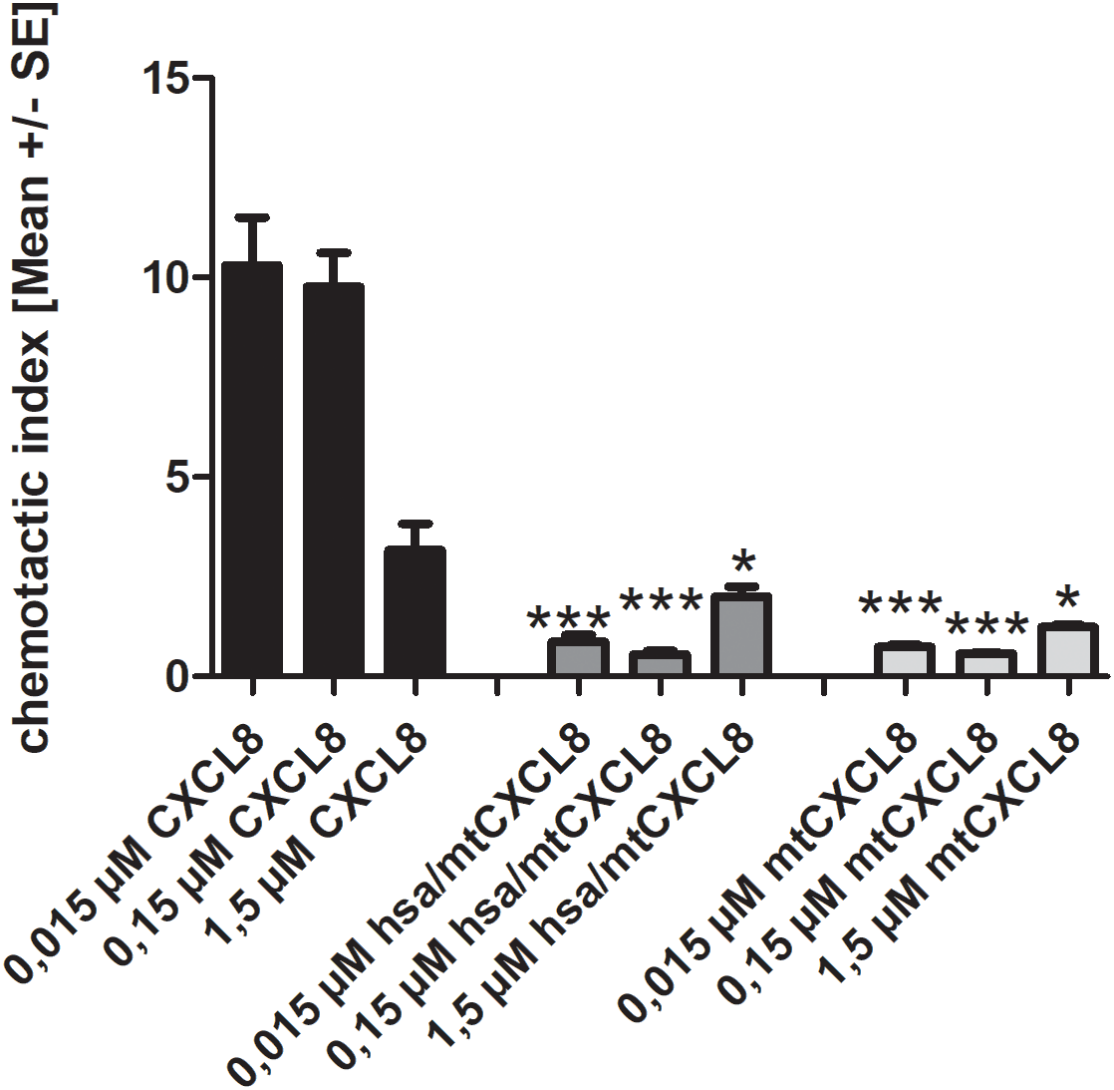
Chemotaxis of human blood-derived neutrophils induced by 1,5/ 0,15 and 0,015 µM CXCL8, mtCXCL8 and hsa/mtCXCL8. Each concentration measured in triplicates on three independent chemotaxis chambers. The chemotactic index is calculated by dividing the migrated cells through background (negative) migration. Thus, a chemotactic index of 1 corresponds to background migration. Student’s Ttest was performed comparing to their respective wt CXCL8 reference concentrations. p<0.5 was considered statistically significant * p<0.05, ** p < 0.01, *** p < 0.001.

In our experiment, we wanted to see if both mtCXCL8 and hsa/mtCXCL8 are equally deficient in mobilizing and attracting neutrophils compared to wildtype CXCL8. Since in both proteins the GPCR activity has been knocked out, a strong reduction of downstream signaling in neutrophils via CXCR1 and CXCR2 has been observed with the Boyden chamber assay (see **Figure 5**): both mtCXCL8 and hsa/mtCXCL8 exhibited a 10-fold reduction of neutrophil migration compared to the wildtype CXCL8 protein at decoy concentrations of 150 and 15 nM. The reduction in neutrophil chemotaxis is almost indistinguishable between the fusion construct hsa/mtCXCL8 and mtCXCL8. This supports the design plan of HSA to being a connecting protein with pharmacological advantages (see below) but with no impact on neutrophil chemotaxis and thus on the potential anti-inflammatory characteristic of the CXCL8 mutant.

The potential of mtCXCL8 and hsa/mtCXCL8 to inhibit neutrophil influx has also been studied in a transmigratory setup (see **Supplementary Figure 2**). Transmigratory assays are designed to investigate the process of transmigration, where immune cells move through endothelial cell layers to reach sites of infection or inflammation. These assays simulate the physiological conditions that immune cells encounter during extravasation, the process by which immune cells migrate from the bloodstream into tissues. In this context, transmigratory assays are particularly valuable for understanding immune cell recruitment and inflammation, key aspects of the body’s response to infection and disease.

In our study, we tested the inhibited migratory response of immune cells using a transwell assay. The assay involves culturing EA.hy 926 cells, a human endothelial cell line, on collagen-coated 96-well Transwell plates to form a barrier that mimics the blood vessel endothelium. These cells were cultured for 48 hours at 37°C with 5% CO_2_, and then stimulated for 4 hours with 50 ng/mL TNFα to induce an inflammatory condition. After this stimulation, neutrophils were collected, as described in the chemotaxis assay section. A total of 1x10^7 stimulated cells were labeled with Calcein AM and then subjected to the migration assay to assess their ability to traverse the endothelial barrier.

In the presence of mtCXCL8 and hsa/mtCXCL8, there was a significant reduction in neutrophil migration through the endothelial layer, as indicated by the fluorescence readings. This suggests that these substances effectively inhibit neutrophil migration, potentially altering the inflammatory response by modulating immune cell recruitment to sites of inflammation. The results highlight the ability of mtCXCL8 and its hsa counterpart to regulate the dynamics of immune cell transmigration, which could have significant therapeutic implications in diseases characterized by excessive or uncontrolled immune cell infiltration, such as in autoimmune diseases or chronic inflammatory conditions.

### 3.5 *In vivo* pharmakocokinetics

To develop a purposeful medication, it is essential to have a substance with a potential long residence time in the blood, so that doses do not need to be administered too frequently. One way to achieve this is to increase the surface area of the substance. In this study, a CXCL8 (mtCXCL8) mutant was coupled to HSA (human serum albumin, hsa/mtCXCL8), and the serum half-life was determined in mice. The rapid degradation of chemokines in the body is a characteristic feature that contributes to the tight regulation of immune responses and inflammatory processes. Proteolytic enzymes, such as metalloproteinases and serine proteases, can cleave and degrade chemokines. This process often occurs in the extracellular environment and is regulated to control the duration and intensity of chemokine signaling.

**Figure 6:**
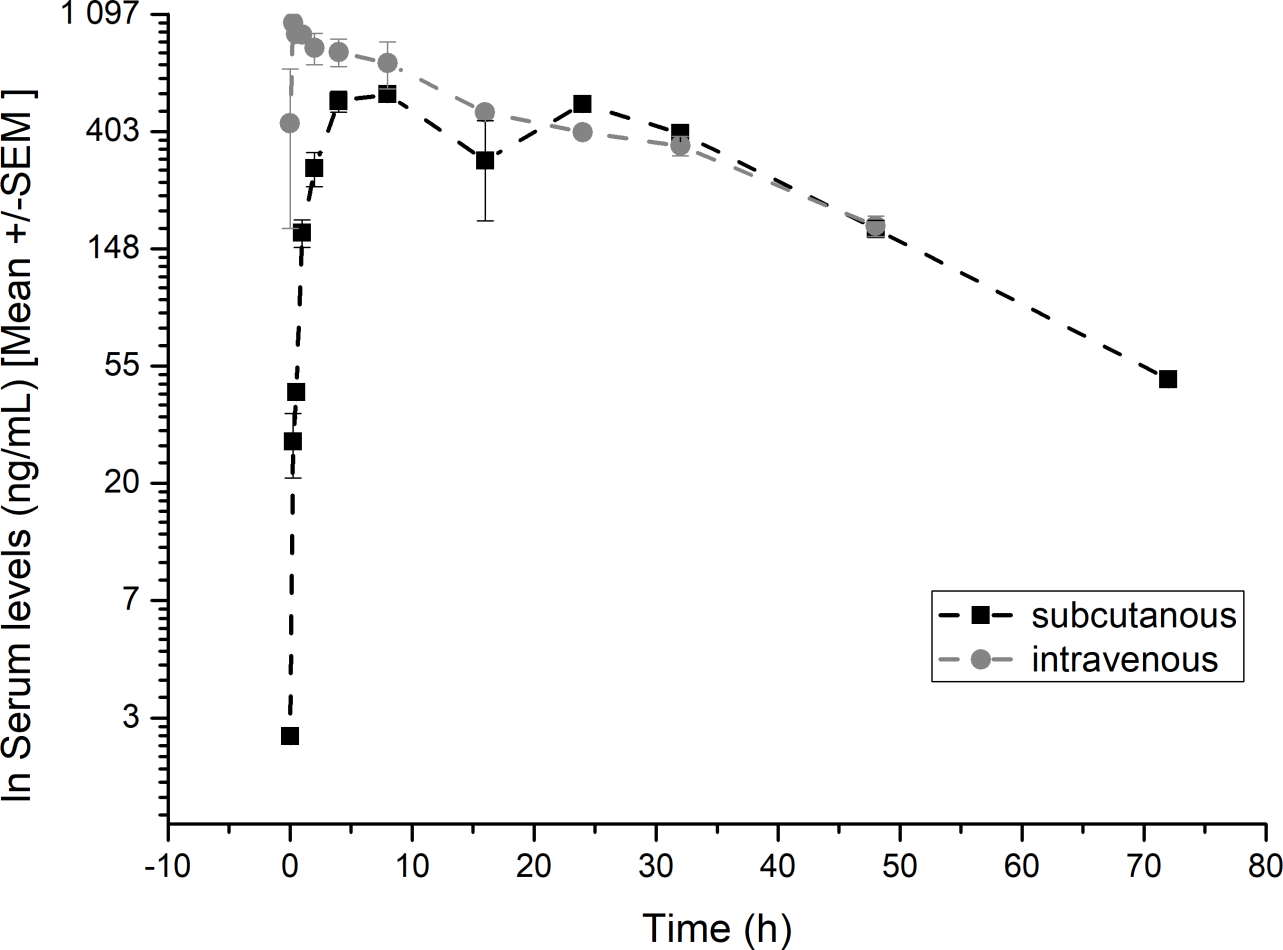
Pharmacokinetic profiles of hsa/mtCXCL8. Balb/CcAnNCrl mice were treated with subcutaneous or intravenous injections of hsa/mtCXCL8 (386 µg/kg body weight). Group size n=3/time point. The serum was analysed using a validated human Serum ELISA kit.

We determined the pharmacokinetic profiles of the hsa/mtCXCL8 in murine serum following subcutaneous and intravenous injection (see **Figure 6**). The first concentration after intravenous injection is probably underestimated since the mice were already anesthetized at the time of administration and were therefore omitted in the analysis.

The serum t1/2 was 22.9 hrs following intravenous and 21.4 hrs after subcutaneous injection, with a mean residence time of 31.7 and 36.5 hours respectively. We have shown in previous studies that Plasma half-life after intravenous administration have been shown to be 10.8 min for wtCXCL8 and 14.3 min for another mtCXCL8 (57).

The hsa/mtCXCL8 also showed excellent bioavailability after subcutaneous administration of 0.99 at 78 hrs.

These results show that the fusion of the CXCL8 mutant with HSA had the desired effect on prolonging exposure of the novel construct. In combination with the high and selective GAG-binding affinity, this innovative construct has the potential to serve as an effective inhibitor of neutrophil migration in clinical applications.

### 3.6 DiscoverX oncology VascHT29 combo ELECT

To mimic *in vitro* the complex tumor and endovascular cell microenvironment, we relied on a commercially supplied and validated test offered by DiscoverX BioMAP^®^. This system uses primary venular endothelial cells, HT-29 colorectal adenocarcinoma cells, and freshly isolated human peripheral blood mononuclear cells (PBMCs), allowing for a physiologically relevant assessment of tumor-host interactions. By integrating these key cell types, the model captures the complex interplay between tumor cells, the vascular network, and infiltrating immune cells, which are essential components of angiogenesis, immune modulation, and tumor progression.

As part of this approach, we selected the Oncology VascHT29 Combo ELECT system, which includes HT-29 colorectal adenocarcinoma cells and venular endothelial cells stimulated for 48 hours via the T-cell receptor Sag, along with PBMCs. This system provides a detailed readout of immune activation, inflammation, and tumor-associated vascular responses, making it an effective tool for evaluating the potential of novel therapeutics in immuno-oncology research.

**Figure 7:**
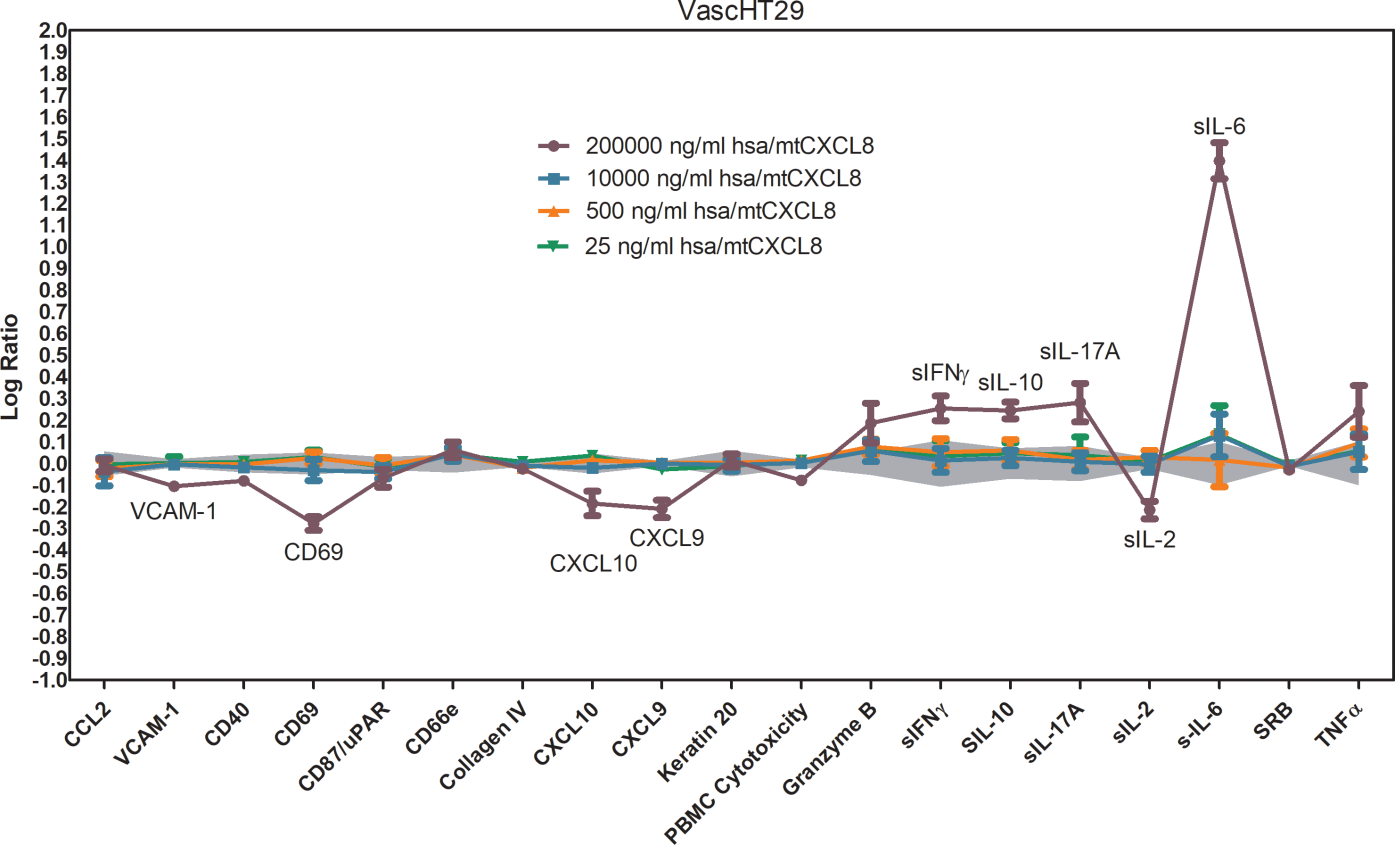
BioMAP Profile of different concentrations of hsa/mtCXCL8 in a VascHT29 System. Relative expression levels (Log ratio Drug/ Vehicle control) for several Protein-based Readout Parameters (Biomakers) Immune-related activities observed for the highest concentration: increased sIL-10, sIL-17A, sIL-6, sIFNγ; decreased CD69, sIL-2. Inflammation-related activities: decreased VCAM-1, CXCL9, CXCL10. Depicted in grey is a 95% significance envelope, generated from historical controls.

Using this system, hsa/mtCXCL8 was found to be non-cytotoxic at all tested doses, supporting its safety profile. At the highest dose, it exhibited immune-related activities by significantly increasing the levels of soluble IL-10, IL-6, IL-17, and IFN-γ, suggesting a role in immune response modulation. It also led to a reduction in soluble IL-2 and CD69, indicating selective regulation of certain immune pathways. In addition, hsa/mtCXCL8 demonstrated inflammatory-related effects by decreasing the expression of VCAM-1, CXCL-10, and CXCL-9, which play key roles in leukocyte adhesion, inflammation, and angiogenesis (**Figure 7**).

Keytruda (pembrolizumab) is an immune checkpoint inhibitor that targets the PD-1 receptor on T-cells, blocking its interaction with PD-L1 and PD-L2, which are often upregulated on tumor cells to evade immune surveillance. By inhibiting this interaction, Keytruda reactivates exhausted T-cells, allowing them to recognize and attack tumor cells more effectively. This leads to increased immune cell infiltration into the tumor, enhancing the anti-tumor immune response. In the Oncology VascHT29 Combo ELECT system, Keytruda (50,000 ng/mL) also reversed the hsa/mtCXCL8-mediated inhibition of key adhesion molecules like VCAM-1, CXCL-10, CXCL-9, and CD69 at the highest dose, promoting immune cell adhesion and recruitment into the tumor site. However, it did not reverse or diminish other effects of hsa/mtCXCL8, suggesting a selective action on specific immune pathways.

**Figure 8:**
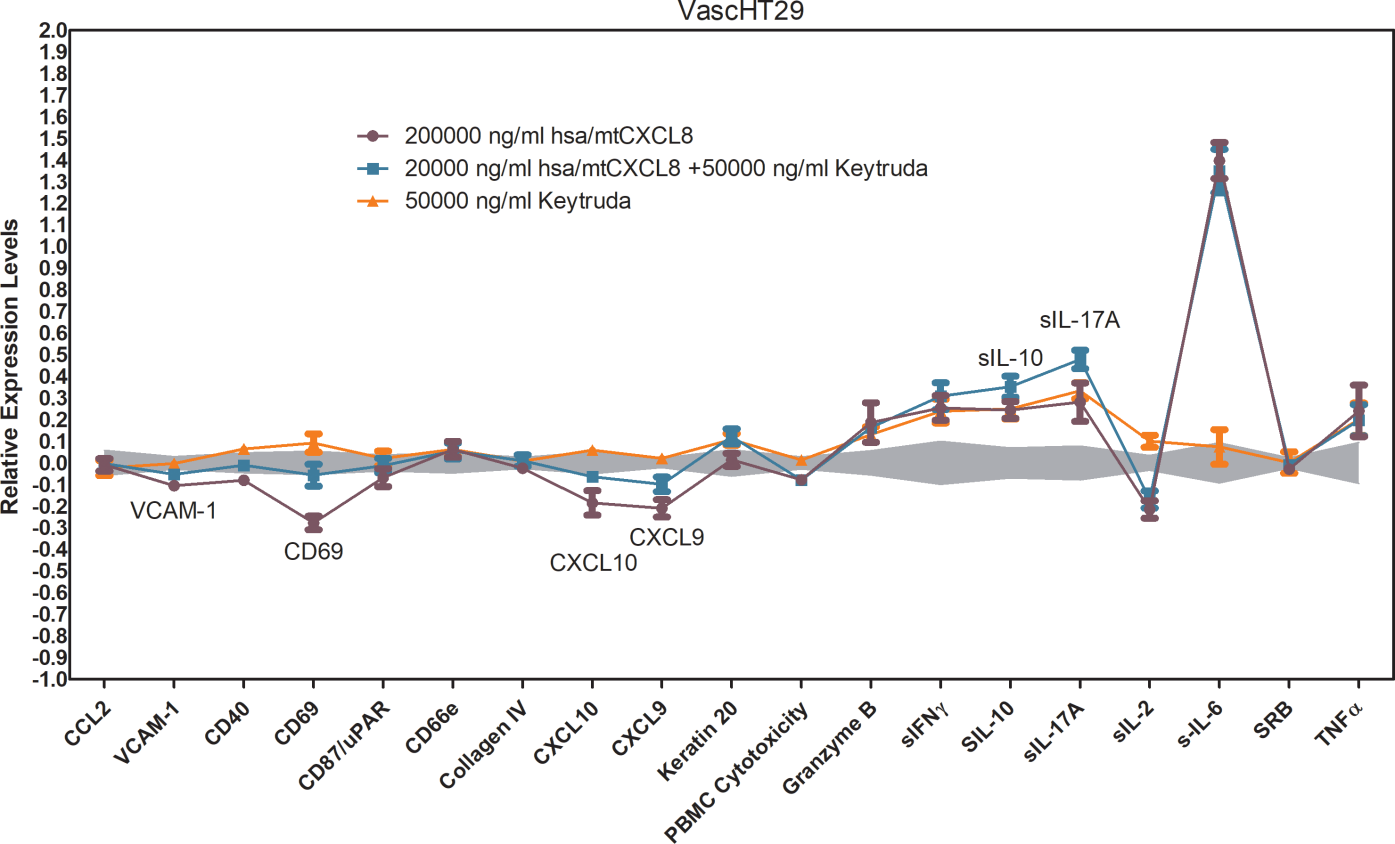
BioMAP Profile of 200 000 ng/ml hsa/mtCXCL8 in combination with 50000 ng/ml Keytruda and the appropriate single condition controls in a VascHT29 System. Relative expression levels (Log ratio Drug/ Vehicle control) for several Protein-based Readout Parameters (Biomarkers). Annotated biomarkers are activities of the combination that are significantly different (p <0.01) than both the individual test agents, and have at least one agent with an effect size of > 20% versus vehicle control. Enhanced upregulation of sIL-10 and sIL-17A indicates enhanced immune-oncology (IO) potential. Reversal of HSA(C34A)-(Gly)_4_Ser-dnCXCL8 mediated inhibition of VCAM-1, CD69, CXCL9 and CXCL10. Depicted in grey is a 95% significance envelope, generated from historical controls.

When hsa/mtCXCL8 is combined with Keytruda, the immune-oncology response is further enhanced. The combination leads to a greater upregulation of soluble IL-10 and IL-17, which suggests that hsa/mtCXCL8 may augment the immune-modulating effects of Keytruda. The increased levels of IL-10 and IL-17 could provide additional benefits in cancer therapy (**Figure 8**). IL-17 is associated with increased T-cell recruitment and tumor immune infiltration, potentially enhancing the ability of immune cells to recognize and kill cancer cells. Meanwhile, IL-10 helps sustain T-cell function and can maintain immune activation without inducing excessive inflammation, supporting a more prolonged and controlled anti-tumor immune response.

The combination of hsa/mtCXCL8 with Keytruda may also improve tumor response by reshaping the tumor microenvironment to become more immune-responsive, amplifying Keytruda’s ability to reactivate T-cells and overcome tumor resistance mechanisms. By using a concentration of has/mtCXCL8 that had no individual effect, we were able to isolate the synergistic impact of the combination and demonstrate that hsa/mtCXCL8 plays a crucial role in modulating the tumor microenvironment to make it more responsive to immune checkpoint blockade. This observation supports the idea that hsa/mtCXCL8 could be leveraged as a complementary treatment to Keytruda, potentially enhancing immune responses and increasing the likelihood of therapeutic success in cancer immunotherapy. The combination of these agents may offer a novel approach to improving treatment outcomes in cancers that are less responsive to single-agent checkpoint inhibition. Taken together, these findings suggest that hsa/mtCXCL8 can enhance immune-oncology responses, potentially making Keytruda more effective in cancer therapy, especially in tumors that are less responsive to checkpoint inhibitors alone.

## 4 Discussion

Glycosaminoglycans (GAGs) constitute a prevalent class of O-linked, highly sulfated polysaccharides that modulate protein activity through interactions with basic amino acids on the target protein (58). Disrupting these functional interactions presents a potential strategy for antagonizing the pathological role of the target protein. Opting for the protein decoy path, we reasoned that if a significant degree of specificity exists between a chemokine and its GAG ligand, CXCL8 could inherently possess specificity for its GAG ligand. While the exact structure of the CXCL-8-specific GAG ligand remains unknown, our approach aimed to leverage the chemokine’s natural ability to recognize its GAG ligand and enhance its affinity, assuming the maintenance of hydrogen bonds and hydrophobic interactions—factors crucial for protein-GAG specificity. This engineering strategy appeared novel and effective for generating potent and selective GAG antagonists at the protein level before (4, 46, 47). Considering their small size, chemokines undergo rapid elimination within the body. To counteract this issue and eliminate the need for frequent dosing of our potential drug and building upon previous studies, we selected one of the CXCL8-based mutants and augmented its size by fusing it to HSA, a strategy previously demonstrated to be successful for a CCL2 decoy (45). When designing hsa/mtCXCL8, potential proteolytic sites have been considered but none was found in the current construct. Human CXCL8 itself is stable in mouse blood (47). Likewise is HSA, and the G_4_S linker does not represent any known proteolytic cleavage site.

As coveted, this fusion not only preserves the GAG-binding affinity but also significantly enhances both the stability and bioavailability of the engineered chemokine *in vivo*. The conformation and GAG ligand binding affinity were mostly unaffected by the attachment of HSA. This stability can be attributed to the potential envelopment of the protein within an HSA pocket, thereby ensuring the preservation of its functional integrity.

The specific fusion protein under examination, hsa/mtCXCL8, emerges as highly promising for applications in conditions where the CXCL8–GAG interaction plays a pivotal role in pathology, such as in chronic inflammation and cancer. The initial *in vitro* studies conducted using the DiscoverX oncology VascHT29 combo ELECT suggest that hsa/mtCXCL8 impacts the immune compartment in this cell model, by modulating cytokine levels and inhibiting immune cells activation markers.

The dramatic increase in soluble IL-6 needs further investigation, given the dual role that IL-6 plays in the tumor microenvironment. Indeed, while IL-6 may support cancer cell proliferation, survival, and formation of metastases, IL-6 signaling may oppose tumor growth by mobilizing anti-tumor T cell immune responses to attempt to control tumor growth and play a key role in the activation, proliferation, and survival of lymphocytes during active immune responses.

When tested in addition to Keytruda, hsa/mtCXCL8 enhanced some of its known activities in this cell model, suggesting beneficial and synergistic effects of a combination therapy.

The limit of the current study is the large dilution range used for hsa/mtCXCL8, and the relatively high concentration required for efficacy. The dose selection was partly due to the known fact that peptides and small proteins may be prone to rapid degradation in the highly active stimulated BioMap system. This will require further consideration of the dose-range selection for future *in vivo* pharmacology models.

Finally, the notable improvements in stability, the prolonged half-life and good subcutaneous bioavailability make this fusion protein a compelling candidate for innovative therapeutic interventions.

## Data Availability

The raw data supporting the conclusions of this article will be made available by the authors, without undue reservation.
