# Supplementary material for: Designing a CXCL8-hsa chimera as potential immunmodulator of the tumor micro-environment
Source: Front Immunol. 2025 Mar 7;16:1539733. doi: 10.3389/fimmu.2025.1539733 (PMC11926544; doi:10.3389/fimmu.2025.1539733)
Supplement: Supplementary file 1 [file DataSheet1.pdf]

Supplemental Material to MS „DESIGNING A CXCL8-HSA CHIMERA AS POTENTIAL IMMUNMODULATOR OF THE TUMOR MICRO-ENVIRONMENT" by Gerlza et al. (MS 1539733)

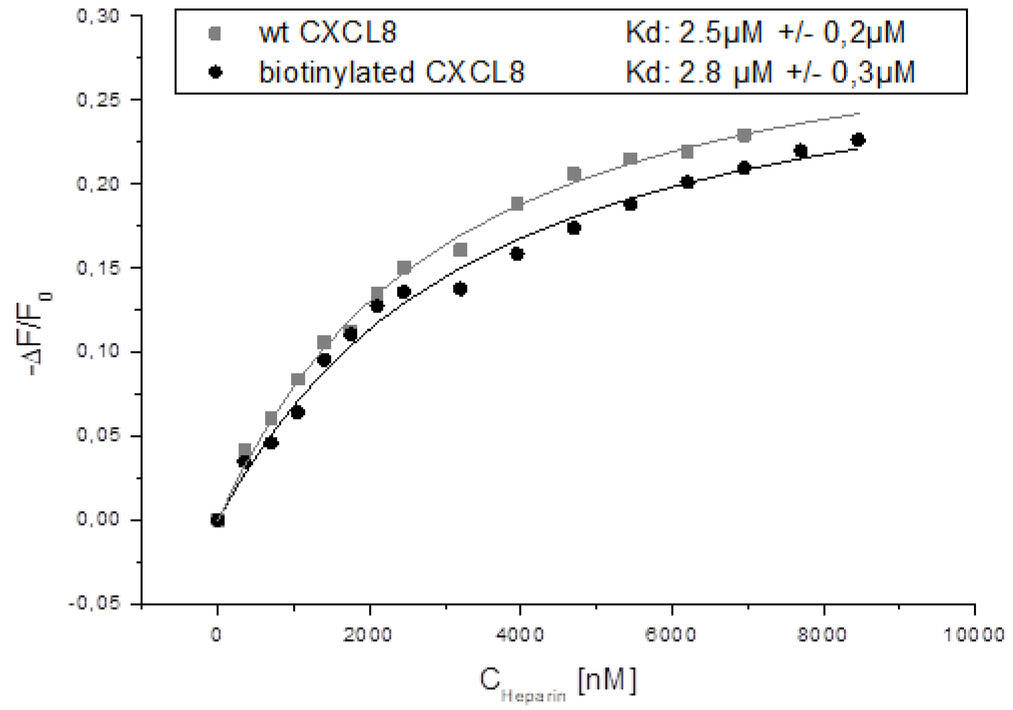

Supplemental Figure 1: Fluorescence binding isotherms for the interaction of wtCXCL8 versus biotinylated CXCL8 with Heparin. The fluctuations in fluorescence are depicted by plotting the normalized change in fluorescence ($-\Delta F/F_0$, with F_0 representing the initial fluorescence intensity before the ligand addition) against the concentration of LMW Heparin. These data are the average values from three separate experiments and were subjected to fitting procedures as detailed in the Method section.

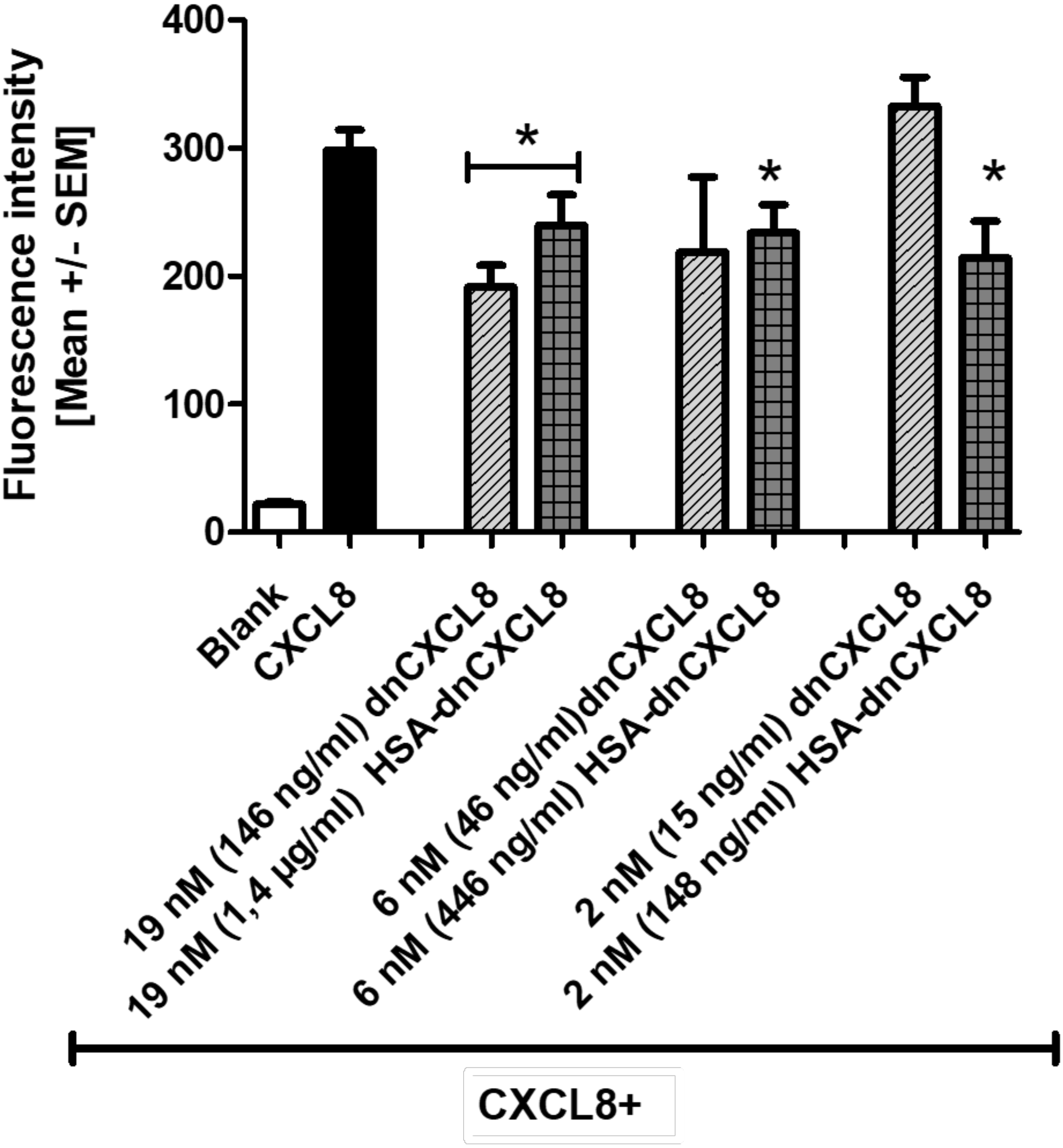

Supplemental figure 2: Transwell setup with human blood-derived neutrophils inhibited with 19/6/2 nM of CXCL8, mtCXCL8 and hsa/mtCXCL8. Student´s t-test was performed comparing to their respective wtCXCL8 reference A p<0.5 was considered statistically significant *
